# Supplementary material for: CD47-SIRPα Signaling Induces Epithelial-Mesenchymal Transition and Cancer Stemness and Links to a Poor Prognosis in Patients with Oral Squamous Cell Carcinoma
Source: Cells. 2019 Dec 17;8(12):1658. doi: 10.3390/cells8121658 (PMC6952929; doi:10.3390/cells8121658)
Supplement: Supplementary file 1 [file cells-08-01658-s001.pdf]

SUPPLEMENTARY INFORMATION

CD47-SIRPα signaling induces epithelial-mesenchymal transition, cancer stemness and links to a poor prognosis in patients with oral squamous cell carcinoma

Disclosed by Shin Pai, MD[1,2], Oluwaseun Adebayo Bamodu, MD., PhD[3,4], Yen-Kuang Lin, PhD[5], Chun-Shu Lin, MD[1,6], Pei-Yi Chu, MD., PhD[7], Ming-Hsien Chien, PhD[1], Liang-Shun Wang, MD., PhD[1,3,8], Hsiao M, PhD[9], Chi-Tai Yeh, PhD[1,3,4,10*], Jo-Ting Tsai, MD., PhD[1, 11,12*]

Authors' affiliation: [1]Graduate Institute of Clinical Medicine, College of Medicine, Taipei Medical University, Taipei City, Taiwan; [2]Department of Oral & Maxillofacial Surgery, Saint Martin de Parres Hospital, Chaiyi City, Taiwan; [3]Department of Medical Research and Education, Taipei Medical University - Shuang Ho Hospital, New Taipei City, Taiwan; [4]Department of Hematology and Oncology, Cancer Center, Taipei Medical University - Shuang Ho Hospital, New Taipei City, Taiwan; [5] Biostatistics Center, Taipei Medical University, Taipei, Taiwan, ROC; [6]Department of Radiation Oncology, Tri-Service General Hospital, National Defense Medical Center, Taipei, Taiwan; [7]Department of Pathology, Faculty of Medicine, Fu Jen Catholic University, New Taipei City, Taiwan; [8]Department of Thoracic Surgery, Taipei Medical University - Shuang Ho Hospital, New Taipei City, Taiwan; [9]Genomics Research Center, Academia Sinica, Taipei, Taiwan; [10] Department of Medical Laboratory Science and Biotechnology, Yuanpei University of Medical Technology, Hsinchu, Taiwan; [11]Department of Radiology, School of Medicine, College of Medicine, Taipei Medical University, Taipei, Taiwan; [12]Department of Radiology, Taipei Medical University - Shuang Ho Hospital, New Taipei City, Taiwan.

*Corresponding author(s):

Chi-Tai Yeh, PhD., Department of Medical Research and Education, Taipei Medical University - Shuang Ho Hospital, New Taipei City 23561, Taiwan; Tel: +886-2-2490088 ext. 8881, Fax: +886-2-2248-0900, E-mail: ctyeh@s.tmu.edu.tw

Jo-Ting Tsai, MD., PhD., Department of Radiation Oncology, Cancer Center, Taipei Medical University - Shuang Ho Hospital, New Taipei City 23561, Taiwan; Tel: +886-2-2490088 ext. 8885, Fax: +886-2-2248-0900, E-mail: 10576@s.tmu.edu.tw

*Title Page (with author names and affiliations)

Working title: Targeting the CD47-CSCs-EMT loop enhances radiosensitivity in OSCC

No.	Target	Dilution	Catalog	kDa
**1**	CD47	1: 1000	B6H12, sc-12730, Santa Cruz	47
**2**	GAPDH	1:500	GAPDH (0411K) Mouse mAb SC-27724	37
**3**	SOX2	1:1000	A-5, sc-365964, Santa Cruz	35
**4**	OCT4	1:1000	C-10, sc-5279, Santa Cruz	45
**5**	CD133	1:1000	MAB4399-1, EMD Millipore	120
**6**	Vimentin	1:1000	D21H3, #5741, Cell Signaling Technology	57
**7**	Slug	1:1000	C19G7, #9585, Cell Signaling Technology	30
**8**	Snail	1:1000	C15D3, #3879, Cell Signaling Technology	29
**9**	N-cadherin	1:500	D4R1H, #13116, Cell Signaling Technology	140
**10**	E-Cadherin	1:500	24E10, #3195, Cell Signaling Technology	135

Supplementary Table S1. Western blot antibodies sheet.

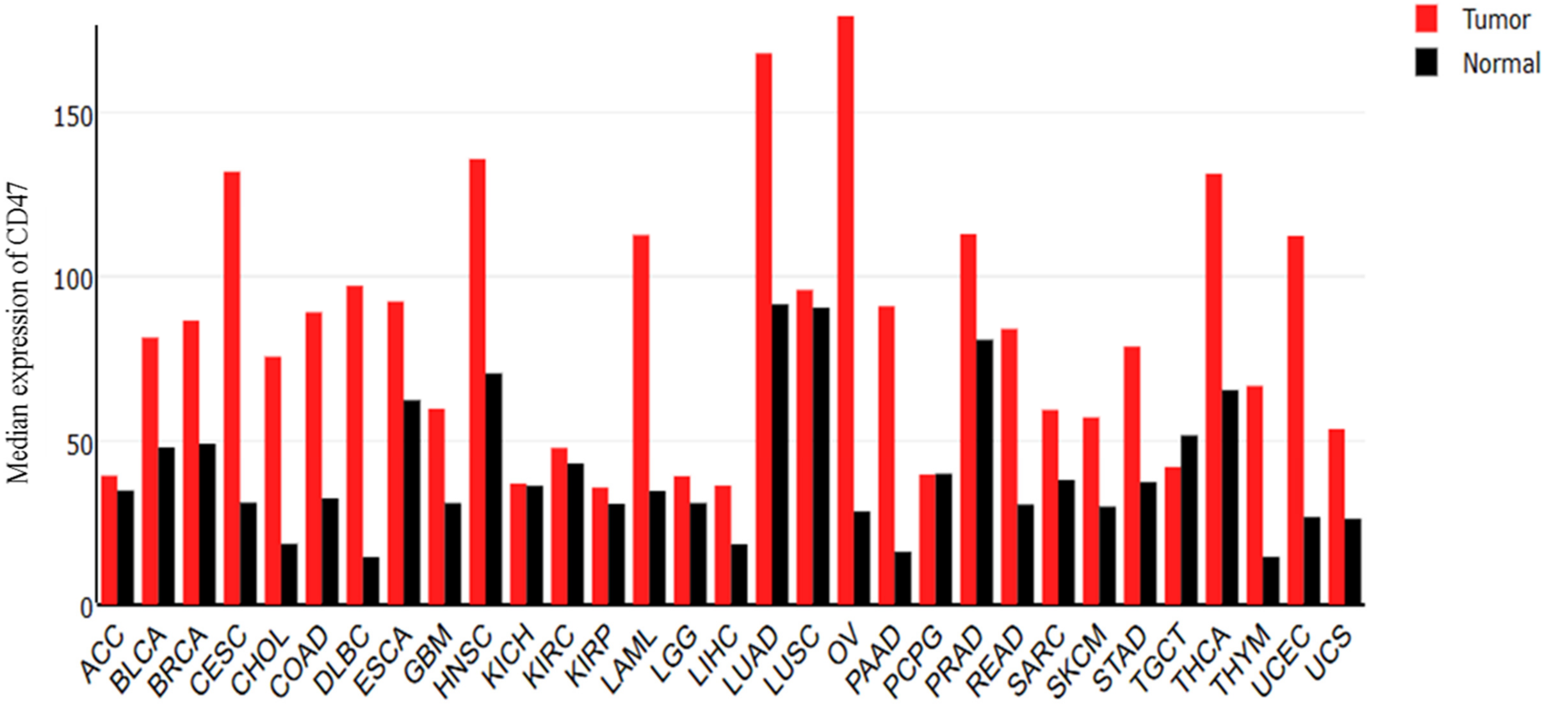

Supplementary Figure S1. CD47 is aberrantly expressed in human oral squamous cell carcinoma. Bar plot of the gene expression profile across all paired tumor samples and normal tissues. The bar height represents the median expression of indicated tumor type or normal tissue.

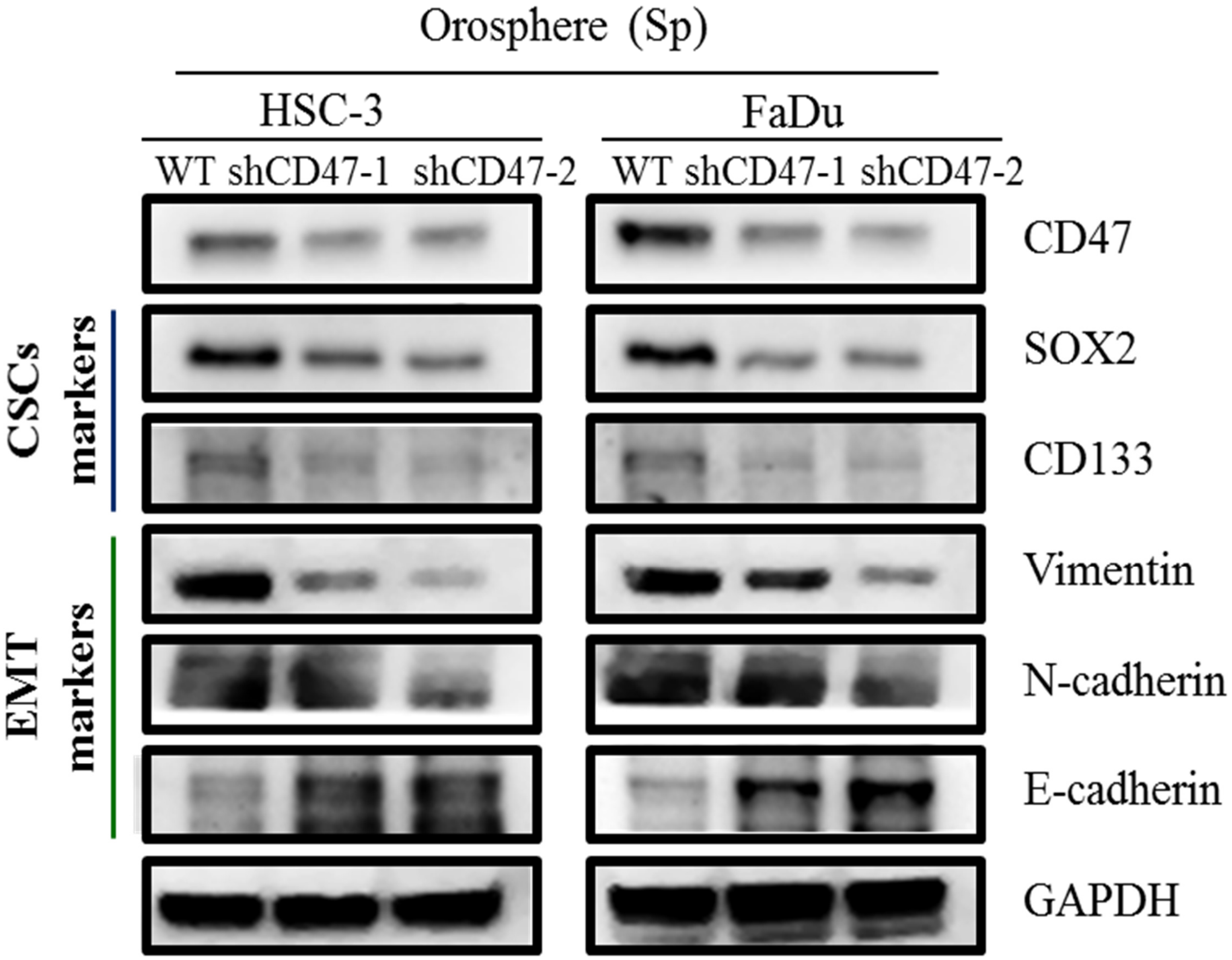

Supplementary Figure S2. CD47 modulates the cancer stem cell-like and metastatic phenotypes of oral squamous cell carcinoma cells. The inhibitory effect of shCD47 on the expression level of CD47, Sox2, CD133, vimentin, N-cadherin, and E-cadherin proteins in HSC-3 and FaDu cells as demonstrated by western blot analyses. GAPDH served as loading control.

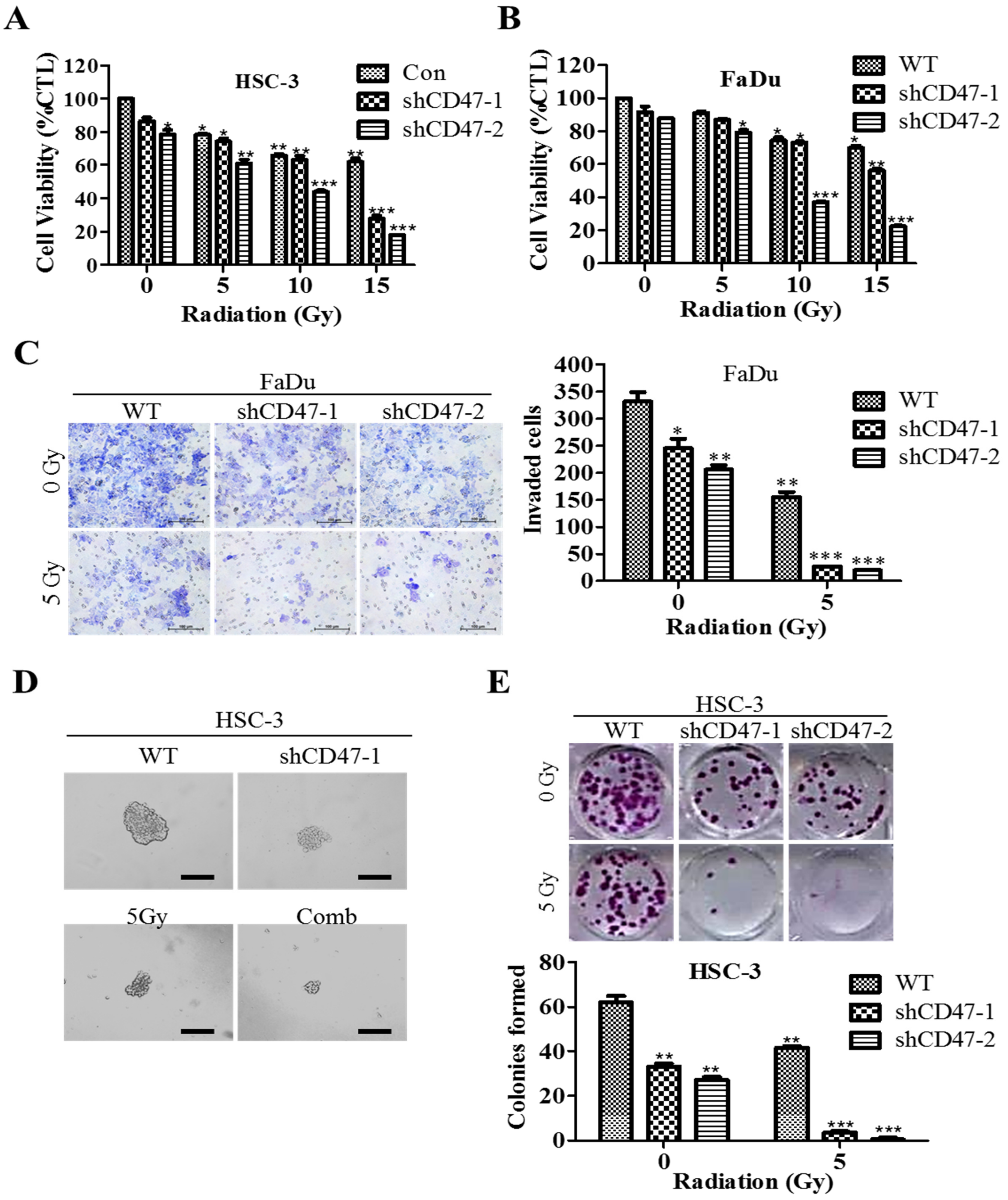

A
HSC-3
Con
shCD47-1
shCD47-2
Cell Viability (%CTL)
Radiation (Gy)
B
FaDu
WT
shCD47-1
shCD47-2
Cell Viability (%CTL)
Radiation (Gy)
C
FaDu
WT
shCD47-1
shCD47-2
0 Gy
5 Gy
Invaded cells
Radiation (Gy)
D
HSC-3
WT
shCD47-1
5Gy
Comb
E
HSC-3
WT
shCD47-1
shCD47-2
0 Gy
5 Gy
Colonies formed
Radiation (Gy)

Supplementary Figure S3. Suppression of CD47 expression enhances the sensitivity of OSCC-SCs to radiation therapy. shCD47 with or without 0 Gy - 15 Gy radiation decreased the viability of (A) HSC-3 and (B) FaDu cells dose-dependently. (C) Transwell invasion assay images show reduced invasion in 5 Gy-exposed shCD47 FaDu cells, compared to their WT counterparts. (D) shCD47-transfected HSC-3 cells exposed to 5 Gy yielded smaller tumorspheres compared to their WT, shCD47, or 5 Gy alone counterparts. (E) shCD47-1 or shCD47-2 HSC-3 cells formed fewer colonies when exposed to 5 Gy, compared to the WT alone cells. *$p<0.05$, **$p<0.01$, ***$p<0.001$

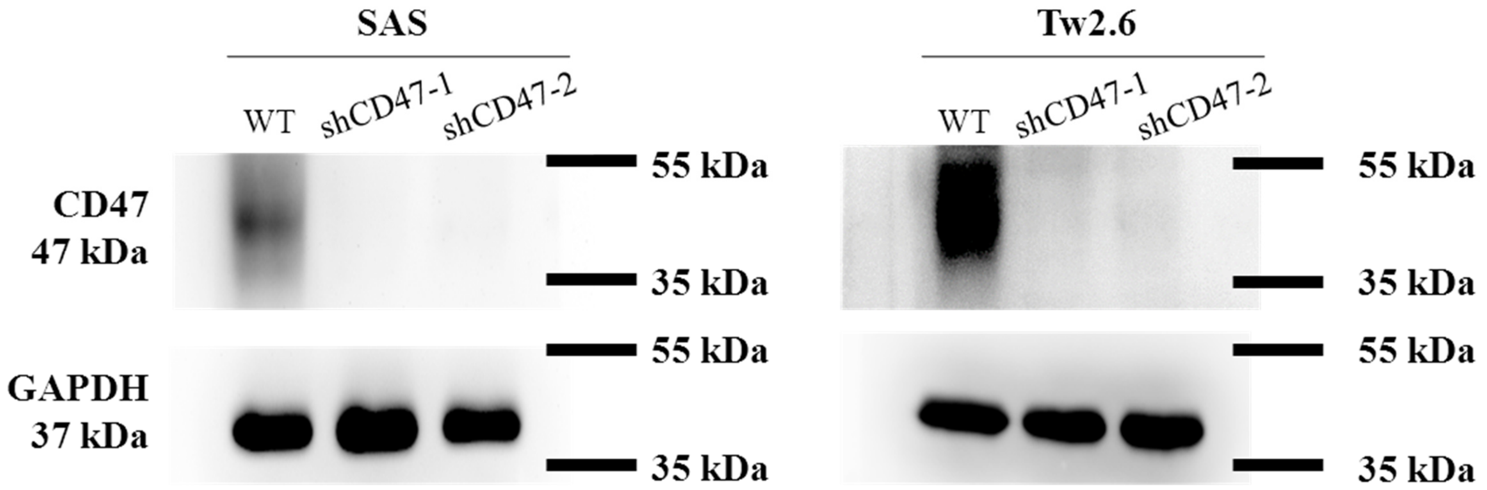

Supplementary Figure S4. Full-size blots of Figure 3B

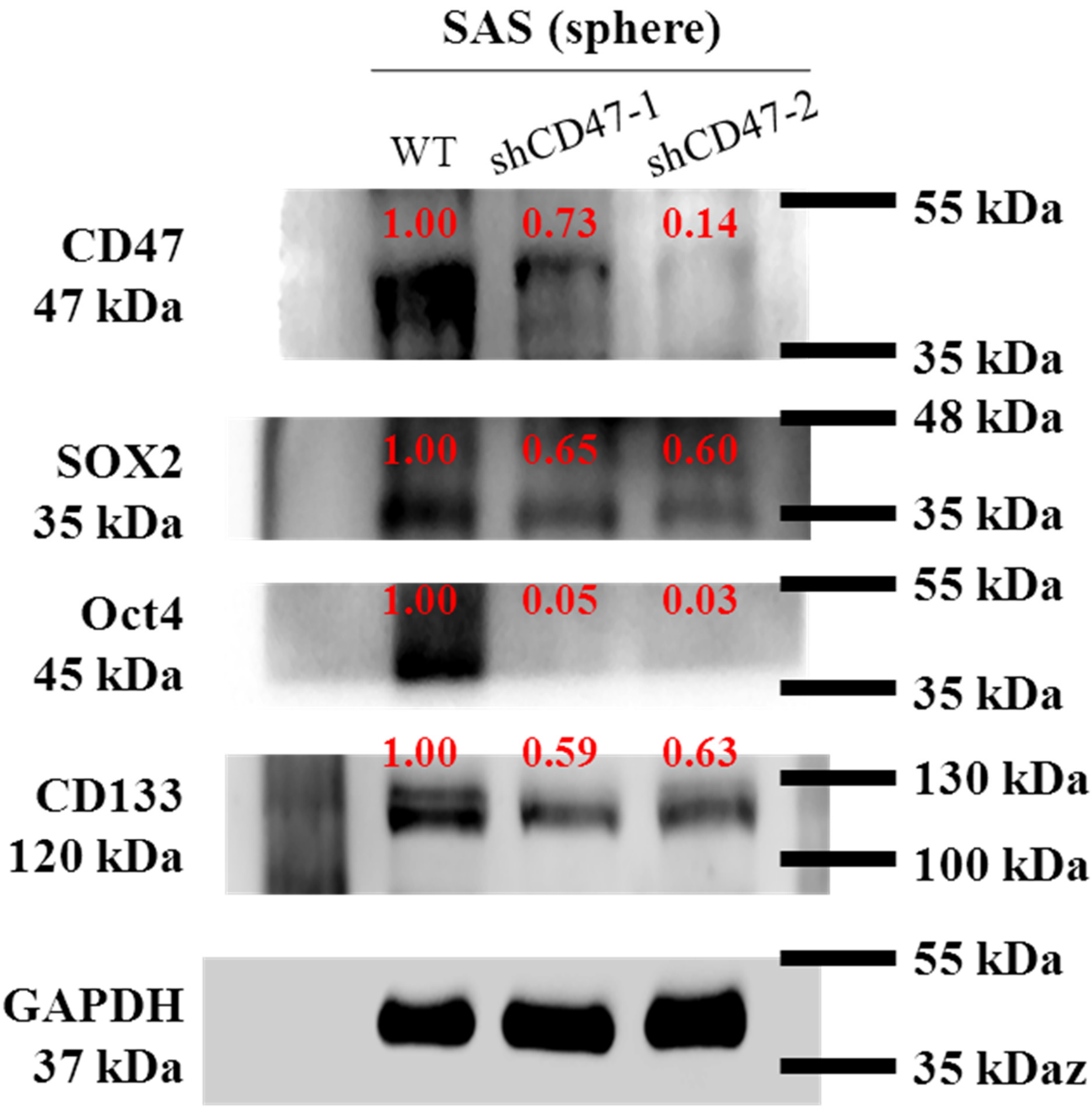

Supplementary Figure S5. Full-size blots of Figure 3C

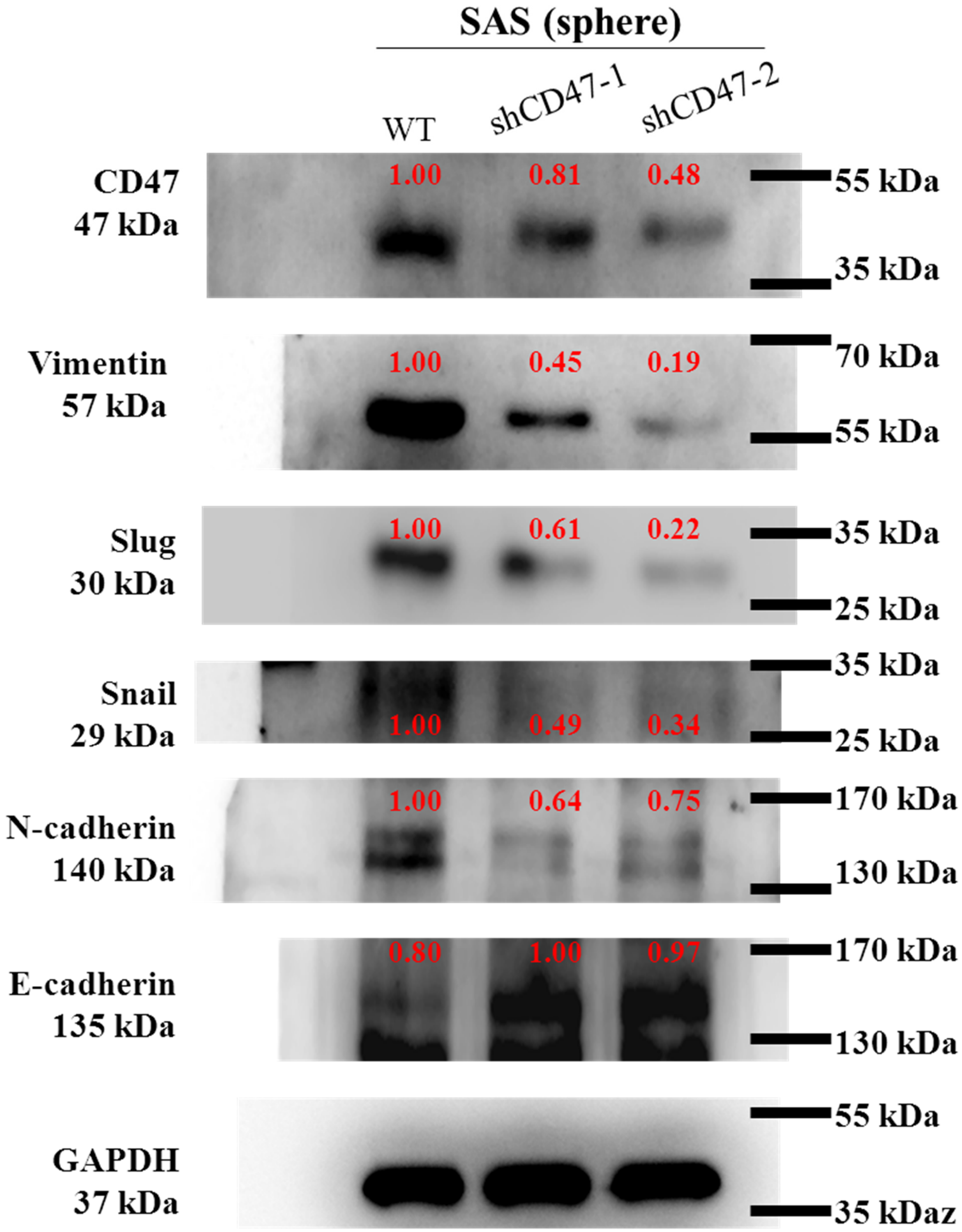

Supplementary Figure S6. Full-size blots of Figure 4D

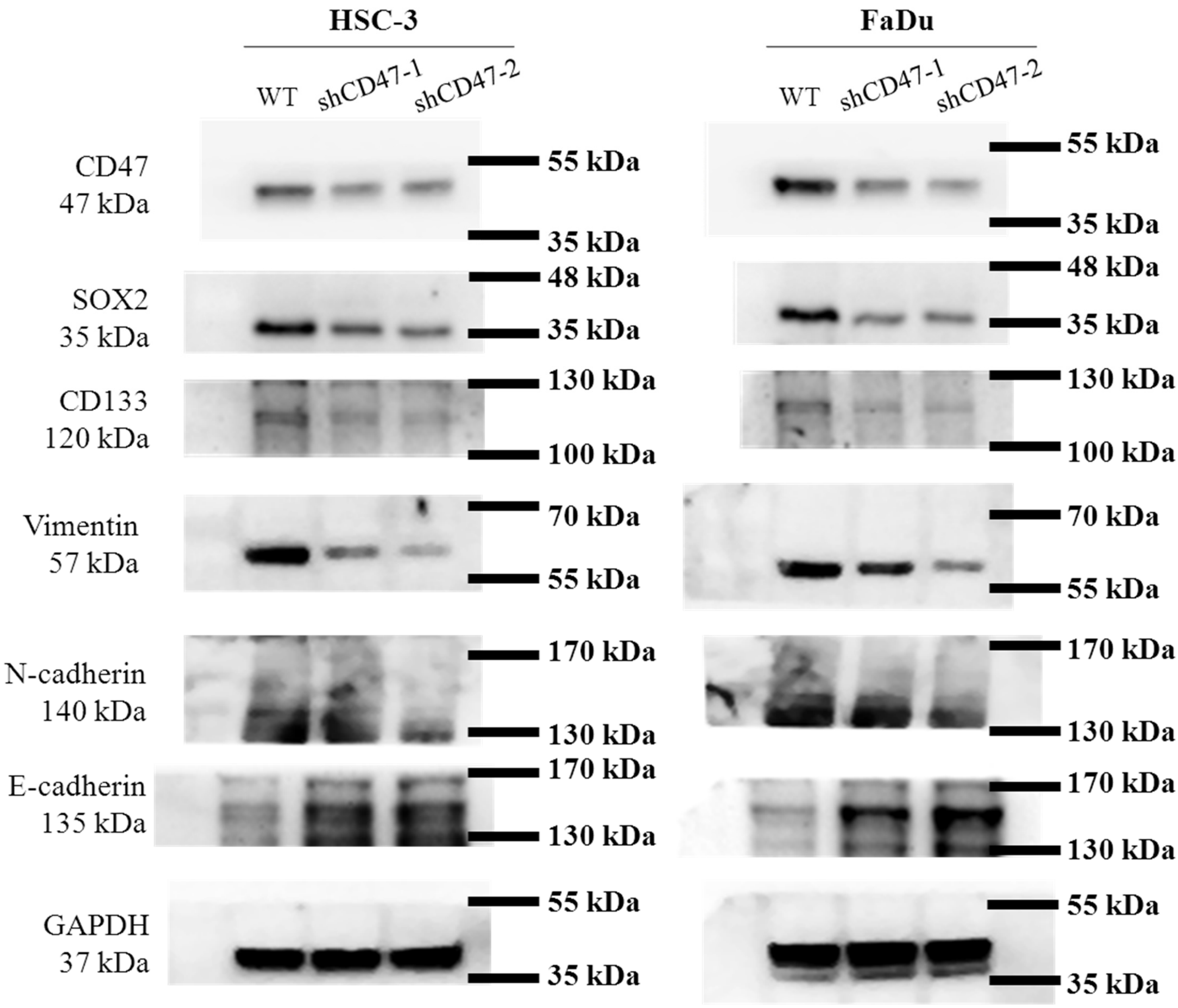

Supplementary Figure S7. Full-size blots of Figure S1
